# Supplementary material for: Topological optical parametric oscillation
Source: Nanophotonics. 2022 Feb 24;11(8):1611–8. doi: 10.1515/nanoph-2021-0765 (PMC11501451; doi:10.1515/nanoph-2021-0765)
Supplement: Supplementary file 1 — Supplementary Material Details [file j_nanoph-2021-0765_suppl.pdf]

# Supplemental Information: Topological Optical Parametric Oscillation

Arkadev Roy, Midya Parto, Rajveer Nehra, Christian Leefmans, and Alireza Marandi*

Department of Electrical Engineering, California Institute of Technology, Pasadena, California 91125, USA

February 1, 2022

*marandi@caltech.edu

# 1 Parametric Oscillation in 1D SSH model

The SSH model consists of N coupled quadratic nonlinear resonators where $\kappa_1 < \kappa_2$. If N is odd, there will be one edge mode, while if N is even there will be two edge modes on either side. The parametric oscillation steady state intensity distribution in both the cases is shown in Fig. S1(a,b).

Now, we derive the condition for bulk instability. For this case, we can assume periodic boundary condition, thereby no existence of edge modes. The linear Hamiltonian in the momentum domain ($k$) is given by:

$$H_L = \begin{bmatrix} 0 & \frac{\kappa_1+\kappa_2 \mathrm{e}^{-\mathrm{i}k}}{2} \\ \frac{\kappa_1+\kappa_2 \mathrm{e}^{\mathrm{i}k}}{2} & 0 \end{bmatrix} \tag{S.1}$$

where, we assume $\kappa_1 = J(1-\epsilon)$ and $\kappa_2 = J(1+\epsilon)$. We assume $J = 1$. All parameters are normalized with respect to $J$. The Bogoliubov- de Gennes Hamiltonian is given by $H_{BdG} = \begin{bmatrix} H_L & H_{NL} \\ H_{NL} & H_L \end{bmatrix}$, where $H_{NL}$ is given by:.

$$H_{NL} = \begin{bmatrix} g/2 & 0 \\ 0 & g/2 \end{bmatrix} \tag{S.2}$$

The eigenvalues determining the dynamical stability of the system is given the eigenvalues of $\sigma_z H_{BdG}$ which can be expressed as:

$$\lambda = \pm\sqrt{\frac{1+\epsilon^2}{2} + \frac{1-\epsilon^2}{2}\cos(k) - \frac{g^2}{4}} \tag{S.3}$$

Therefore, bulk instability will occur for $g > 2\epsilon$, when the eigenvalues will be imaginary. Such a distribution of eigenvalues in such a scenario is given in Fig. S1(c,d).

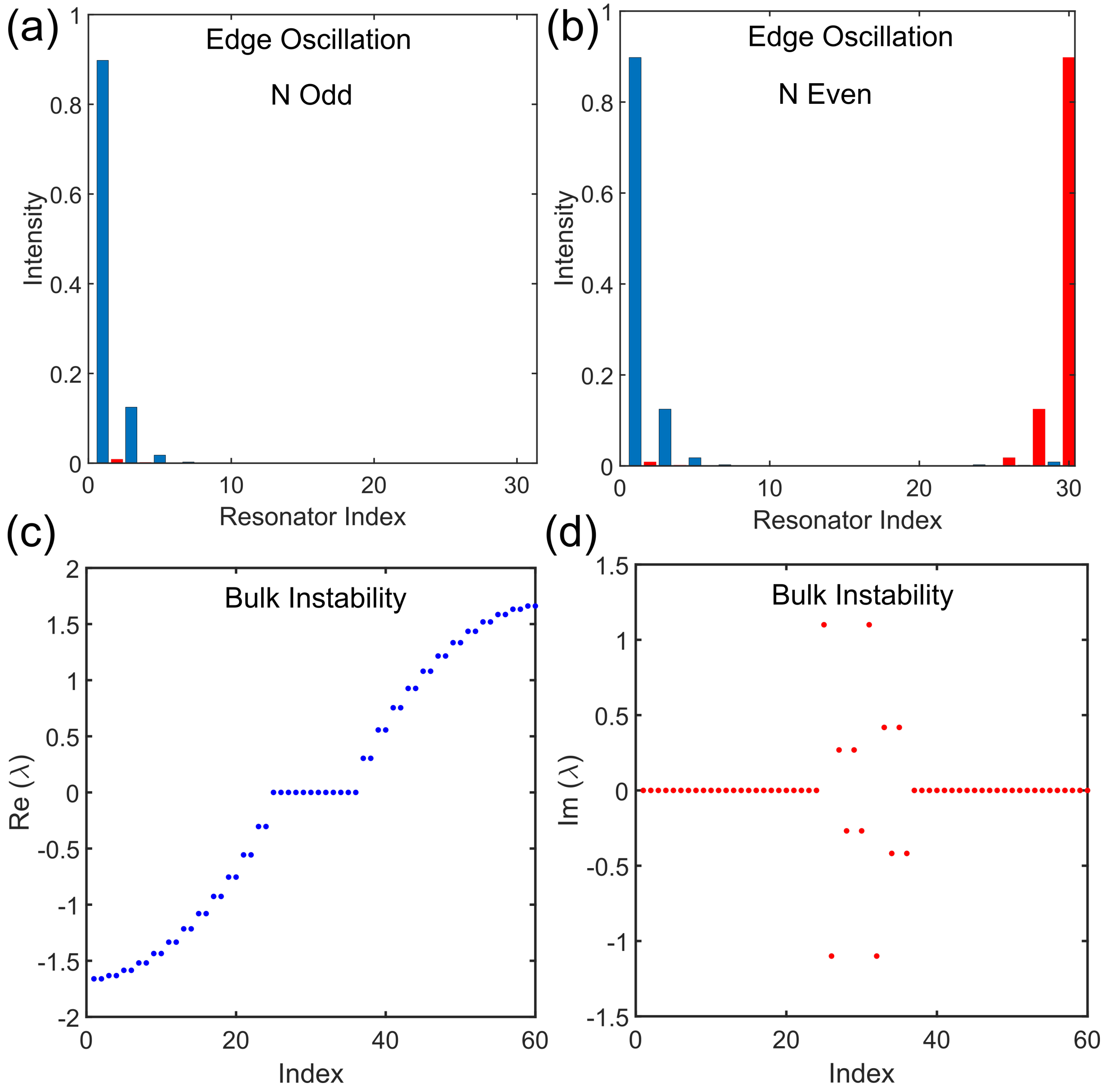


Figure S1: Parametric oscillation in the edge mode for 1D SSH model when a) N is odd and b) N is even. Eigenvalue diagram when bulk instability occurs c) the real part and d) the imaginary part. Parameters used in the simulation are: $J = 1, \epsilon = 0.5, g = 1.1$, and $N = 30$.

# 2 Parametric oscillation in 2D Lattice :

## 2.1 Topologically trivial case :

We consider a scenario where the gauge field is zero in the 2D Kagome lattice. This is the topologically trivial case. In this case, in the presence of quadratic parametric interactions bulk instability will occur. The band diagram is shown in Fig. S2(a,b). The intensity distribution of the OPOs arranged in the lattice is shown in Fig. S2(c), showing the oscillation occurs throughout the lattice.

The time evolution of the buildup of the intensity of the OPOs in the lattice in this case can be found here: Video1.

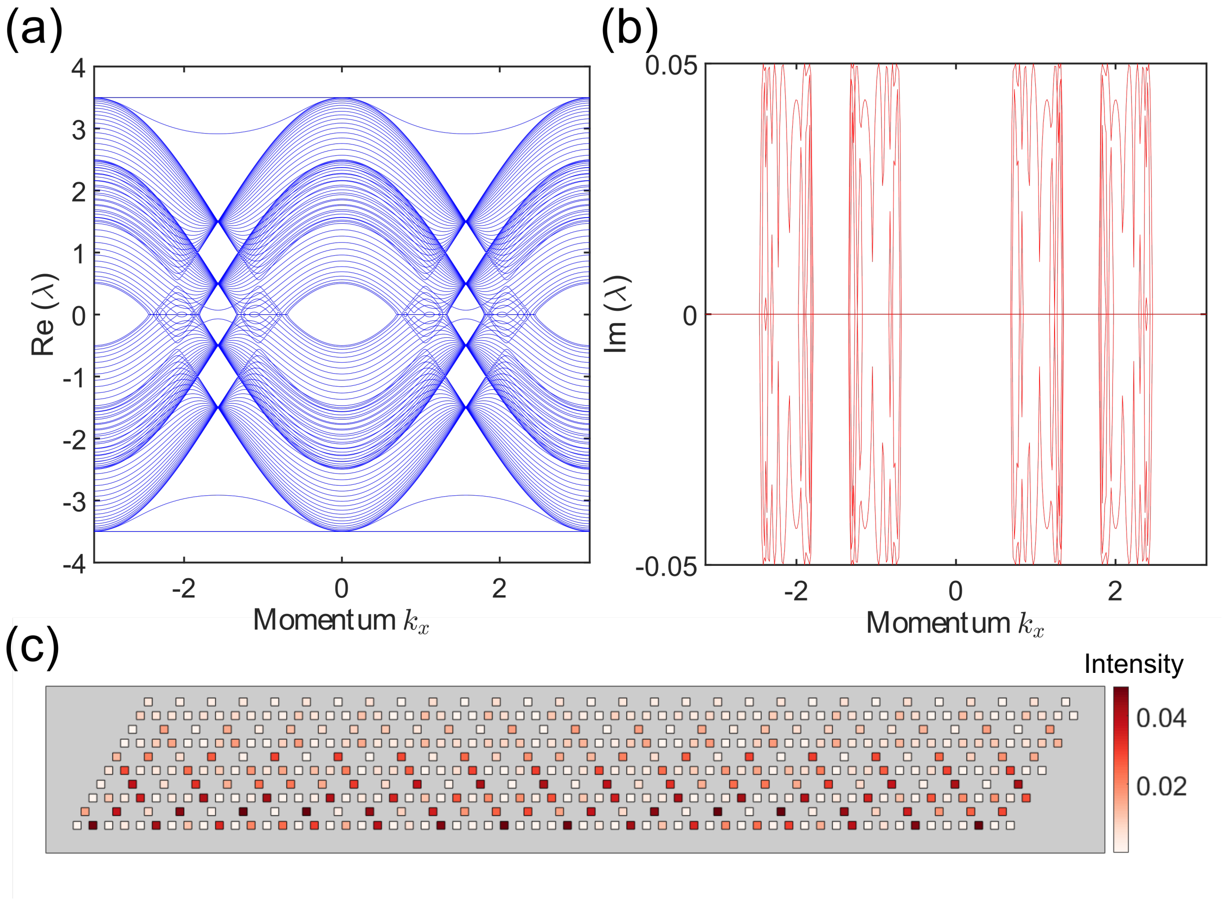


Figure S2: **Parametric oscillation in the topologically trivial case.**. Band diagram of the Kagome lattice strip with a) showing the real and b) the imaginary part of the eigenvalues. c) The intensity distribution of the OPOs in the lattice in the topologically trivial case.

## 2.2 Topologically non-trivial case :

In the presence of gauge field, and the appropriate parameters we obtain topologically protected chiral edge states as described in the main text. We are considering the non-positive definite case of the bosonic

pairing Hamiltonians [1]. Here the particle and hole bands cross each other. The particle bands are identified as: $V^{\dagger}\sigma_z V > 0$, and the hole bands as: $V^{\dagger}\sigma_z V < 0$, where $V$ is the eigenvector. The band diagram with the particle and hole bands are shown in Fig. S3(a)

In the presence of a defect in the form of a missing lattice unit cell, the edge is modified around the defect, but still the chirality is retained in the topologically non-trivial case.The intensity distribution of the OPOs arranged in the lattice is shown in Fig. S3(b), showing the oscillation occurring along the edge surrounding the defect.

The time evolution of the buildup of the intensity of the OPOs in the lattice in this case with a defect can be found here: Video2

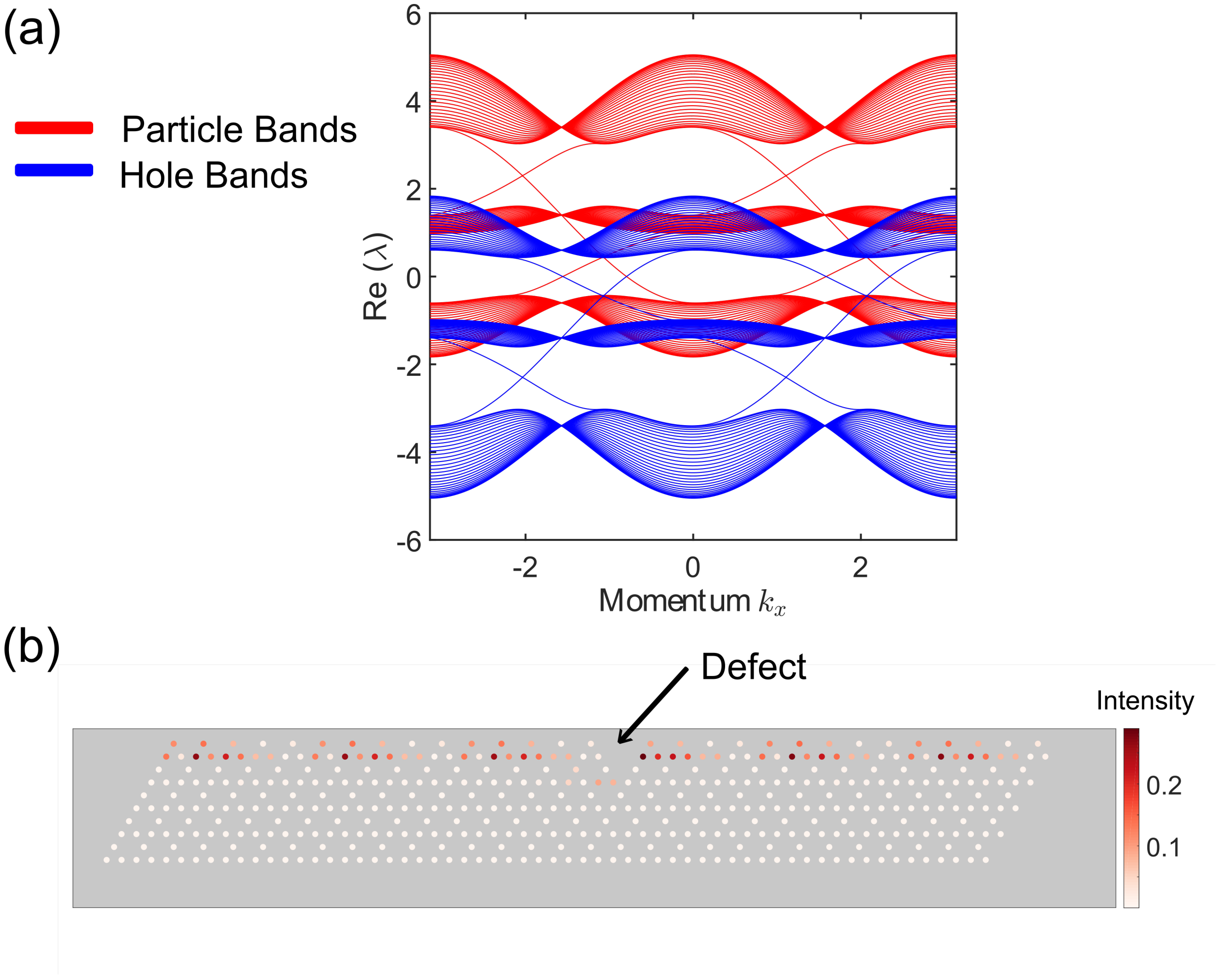


Figure S3: a) Particle and the hole bands in the band-diagram. Parameters used in the simulation are $\Delta = 1.4, \phi = 0.6\pi, g = 0, J = 1$. b) The intensity distribution of the OPOs in the lattice in the topologically non-trivial case in the presence of a defect.

The time domain evolution of the intra-cavity intensity of an OPO lying on the edge is shown in Fig. S4(a,b). The spectral domain power distribution is shown in Fig. S4(c). The intra-cavity field of the OPOs on the edge are phase locked with respect to each other.

The time evolution of the buildup of the intensity of the OPOs in the lattice in the non-trivial case can be found here: Video3. It features chiral propagation. The direction of propagation can be reversed by changing the sense of hopping phase per plaquette to clockwise as shown here: Video4.

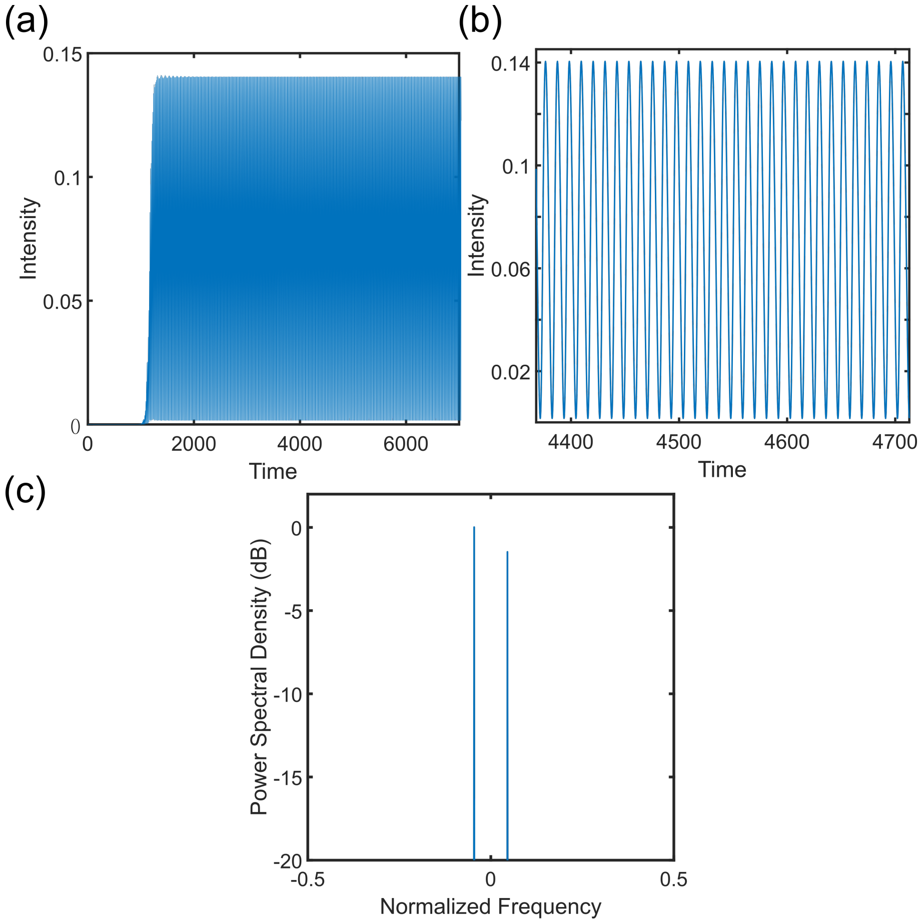


Figure S4: a) Time domain evolution of the intra-cavity intensity of an OPO lying on the edge in the 2D Kagome lattice. b) The zoomed in view of (a). c) Power spectral density. Parameters used in the simulation are: $\Delta = 1.4, \phi = 0.6\pi, g = 0.015, J = 1, \alpha = 0.002, g_s = 0.1$

# 3 1D SSH lattice in the quantum regime:

In order to compare the protection of squeezed quantum state from coupling disorders between topologically non-trivial and trivial case, we consider the following. We consider a topologically trivial SSH lattice where $\kappa_1 > \kappa_2$. We select the gain parameter such that one mode undergoes instability (non-zero imaginary part). This is shown in Fig. S5. The eigenvector is mostly localized in the Nth resonator. We compare the quadrature squeezing i this resonator with the edge OPO of the topologically non-trivial case in the main text. Both the cases are taken to be equal times below the oscillation threshold, and same out-coupling efficiency.

The squeezed quantum state in the 1D SSH model is robust to coupling disorders, when the coupling

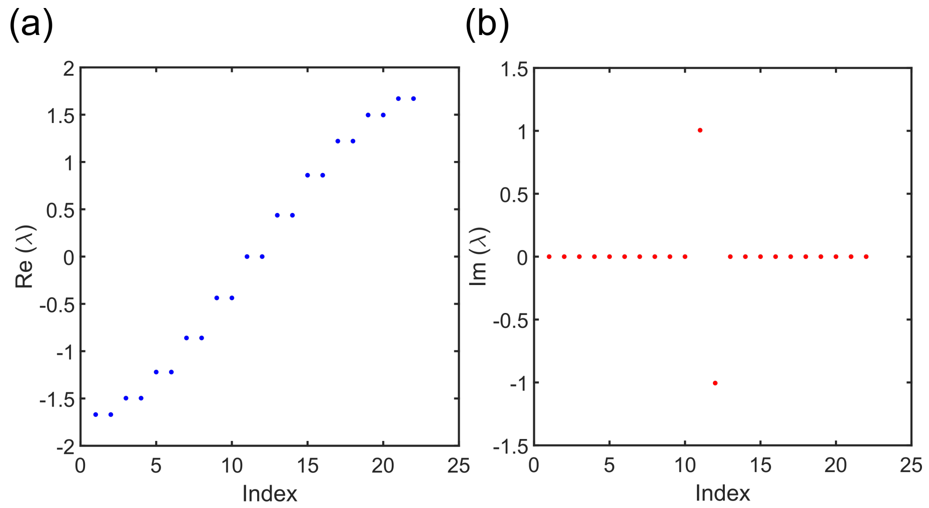


Figure S5: Eigenvalue diagram for topologically trivial SSH lattice with gain experienced by a single mode. a) the real part . b) the imaginary part. The parameters used in the simulation are : $g = 1.005, \alpha = 1.206, \rho = 0.9, \epsilon = 0.5, J = 1, N = 11$.

disorder strength is small compared to the bandgap. However, it is not protected from the detuning induced disorder. The detuning disorder doesn't preserve the chiral symmetry of the SSH model. In the presence of detuning disorder the optimum squeezed quadrature gets rotated and the squeezing is degraded due to the influence of the anti-squeezed quadrature. Fig. S6(a) shows that the squeezed quantum state is susceptible to detuning induced disorder.

When we ensure that the edge mode only experience gain, and all other modes are stable. This causes a gap in the imaginary part of the eigenvalue spectrum. With the increasing value of the gain parameter ($g$), this gap increases. Increasing this gap results in less deleterious effect of the detuning disorder. The dependence of the degradation of squeezing in the presence of detuning disorder as a function of the gain parameter is shown in Fig. S6(b). The errorbar indicates the standard deviation of the squeezing variation for each gain parameter value where 5 sets of 100 realizations of disorder are considered. However, this is not a topological effect.

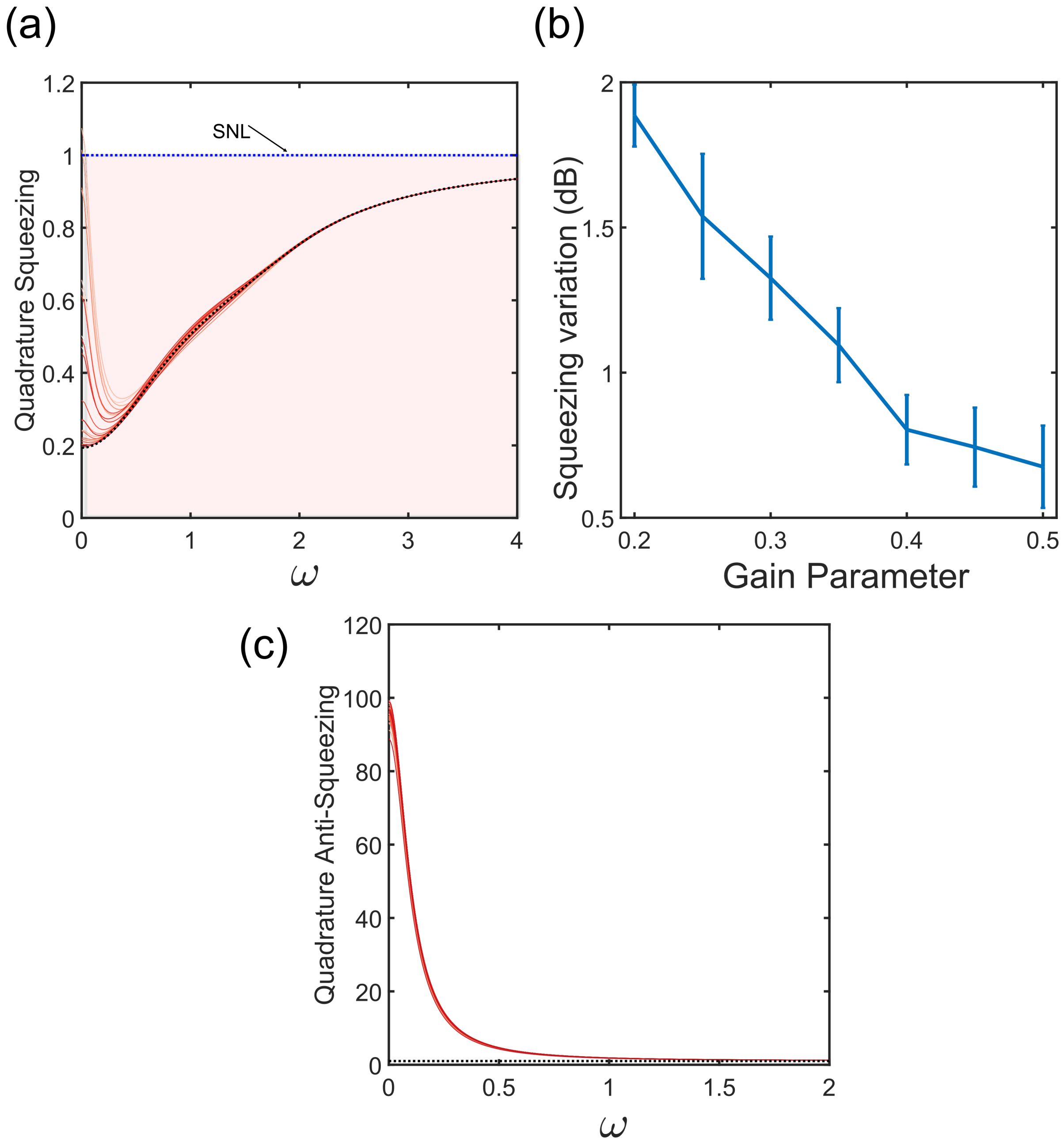


Figure S6: a) Absence of robustness in the quadrature squeezing for the edge OPO, in the presence of detuning disorder. Multiple squeezing spectra corresponding to different disorder realizations are overlaid on top of each other. The black dotted line refers to the squeezing in the absence of detuning and coupling disorder. SNL refers to the shot noise limit. The parameters used in the simulation are: $g = 1, \alpha = 1.2, \rho = 0.9$(out-coupling efficiency)$, \epsilon = 0.5, J = 1, N = 11$. b) effect of the eigenvalue gap (in the imaginary component of the eigenvalue) on the quadrature squeezing degradation in the presence of detuning disorder. The gap is varied by changing the gain parameter ($g$). The disorder is assumed to be normally distributed with standard deviation of 0.05. c) The effect of coupling disorder in the anti-squeezed quadrature.

## 4 Model of the OPO in the classical regime:

We model the OPO using a simplified governing equation which takes into account the parametric gain and the gain saturation (due to second harmonic generation of the signal back to the pump). We assume the OPO to operate at degeneracy. The parametric oscillation occurs in a high Q cavity, and is oscillating in a CW mode.

**Non-resonant pump configuration**

The quadratic nonlinear interaction happening in the phase matched $\chi^{(2)}$ region is given by:

$$\frac{da}{dz} = \epsilon b a^* \tag{S.4a}$$

$$\frac{db}{dz} = -\frac{\epsilon}{2} a^2 \tag{S.4b}$$

where, $a$ and $b$ represents the signal and pump field envelopes respectively. $\epsilon$ is the effective nonlinear co-efficient. Let, $L$ be the length of the nonlinear interaction region. In the high Q limit, we can assume that the field does not change significantly within a round-trip. So, Eq (S.4b) can be expressed in the $n^{th}$ round-trip as:

$$b_n = b_0 - \frac{\epsilon}{2} L a_n^2 \tag{S.5}$$

where, $b_0$ is the input pump. We can express the evolution of the signal field on a round-trip basis as:

$$a_{n+1} = a_n + \epsilon b_0 L a_n^* - \frac{1}{2}\epsilon^2 L^2 |a_n|^2 a_n \tag{S.6}$$

The loss in each round-trip can be included as:

$$a_{n+1} = a_n e^{-\gamma T} \tag{S.7}$$

The detuning in each round-trip can be included as:

$$a_{n+1} = a_n e^{\mathrm{i}\Delta T} \tag{S.8}$$

where, $\Delta T$ is the total detuning per round-trip. where, $\gamma$ is the loss per unit time, and $T$ is the round-trip time. In the high Q limit, we can convert the difference equation in a differential form as:

$$T\frac{da}{dt} = -\gamma T a + \mathrm{i}\Delta T a + \epsilon b_0 L a^* - \frac{1}{2}\epsilon^2 L^2 |a|^2 a \tag{S.9}$$

This reduces to:

$$\frac{da}{dt} = -\gamma a + \mathrm{i}\Delta a + \epsilon b_0 \frac{L}{T} a^* - \frac{1}{2T}\epsilon^2 L^2 |a|^2 a \tag{S.10}$$

Thus, we define $g = \epsilon b_0 \frac{L}{T}$ which denotes the parametric gain, and $g_s = \frac{1}{2T}\epsilon^2 L^2$ which denotes the gain saturation co-efficient.

The dynamics of a single degenerate OPO is governed by the following simplified equation:

$$\frac{da}{dt} = -\alpha a + \mathrm{i}\Delta a + g a^* - g_s |a|^2 a \tag{S.11}$$

where, $a$ is the amplitude of the intra-cavity field, $\alpha$ is the loss parameter (arising due to propagation loss/ out-coupling loss), $\Delta$ represents the detuning parameter (detuning between the subharmonic $\omega$ and

the resonance frequency of the cavity), $g$ represents the parametric gain that depends on the effective quadratic nonlinearity and the pump power, and $g_s$ is the gain saturation nonlinearity co-efficient. The gain saturation arises due to the pump depletion.

The lattice of OPOs represents coupled OPOs, where the coupling is determined by the linear part of the Hamiltonian.

# 5 Model of the coupled OPO system (SSH Lattice) in the quantum regime: Langevin equations

Here we develop the input-output formalism of the coupled OPO system for analysis of the quantum behavior. This formalism has been used to obtain the results of the Quantum Regime subsection of the Results section of the main text.

The signal fields $\hat{a}_i, i \in 1 \ldots N$ (where $N$ is the number of OPOs in the finite SSH lattice) in the resonators constituting the OPO's experience a roundtrip loss ($\gamma$) consisting of two contributons: out-coupling loss ($\mu$) and round-trip propagation loss ($\alpha$). We define ($\rho$) as, $\rho = \frac{\mu}{\gamma}$ , which is the ratio between out-coupling and total loss. The OPOs operate below threshold. Also the pump is non-resonant and the pumps driving the OPO's are not coupled to each other. So we can adiabatically eliminate the pump dynamics and represent the pump field with a coherent field. We assume all OPOs experience identical loss, coupling, and pump drives.

We include the loss arising from different mechanisms and the accompanying fluctuations ($\hat{V}$) using the input-output formalism of open quantum systems. We can write the Heisenberg-Langevin equations for the intra-cavity field as [2]:

$$\dot{\hat{a}}_i = -\gamma \hat{a}_i + g\hat{a}_i^\dagger + \sum_{j \neq i} \mathrm{i}\kappa_{i,j}\hat{a}_j + \sqrt{2\alpha}\hat{V}_{\alpha,a_i} + \sqrt{2\mu}\hat{V}_{\mu,a_i} \tag{S.12a}$$

where, $\kappa_{i,j} = H_L(i,j)$ is the (i,j) the term of the linear Hamiltonian ($\hat{H_L}$) given in Eq(1) of the main text expressed in the matrix (NxN) form.

The respective output field can be mapped to the intra-cavity field as:

$$\hat{A}_{i,out} = \sqrt{2\mu}\hat{a}_i - \hat{V}_{\mu,a_i} \tag{S.12b}$$

We assume that the fluctuations have zero mean field and is delta-correlated Gaussian noise sources. Noise from independent channels are non-correlated. These fluctuations which provides the Langevin forces follow the following commutation relations:

$$\begin{gathered} \left\langle \hat{V}_{l,j}(t)\hat{V}^\dagger_{l'j'}(t^{'})\right\rangle = \delta_{ll'}\delta_{jj'}\delta(t-t^{'}) \\ \left\langle \hat{V}^\dagger_{l,j}(t)\hat{V}_{l'j'}(t^{'})\right\rangle = \left\langle \hat{V}_{l,j}(t)\hat{V}_{l'j'}(t^{'})\right\rangle = \left\langle \hat{V}^\dagger_{l,j}(t)\hat{V}^\dagger_{l'j'}(t^{'})\right\rangle = 0 \end{gathered} \tag{S.13a}$$

where $l \in \{\mu, \alpha\}$ and $j \in a_i, i \in 1 \ldots N$. We define the Fourier transform as: $\tilde{V}(\omega) = \frac{1}{\sqrt{2\pi}}\int_{-\infty}^{\infty} dt\hat{V}(t)e^{i\omega t}$. Therefore in the spectral domain the noise correlations appear as:

$$\begin{gathered} \left\langle \tilde{V}_{l,j}(\omega)\tilde{V}^\dagger_{l'j'}(\omega^{'})\right\rangle = \delta_{ll'}\delta_{jj'}\delta(\omega-\omega^{'}) \\ \left\langle \tilde{V}^\dagger_{l,j}(\omega)\tilde{V}_{l'j'}(\omega^{'})\right\rangle = \left\langle \tilde{V}_{l,j}(\omega)\tilde{V}_{l'j'}(\omega^{'})\right\rangle = \left\langle \tilde{V}^\dagger_{l,j}(\omega)\tilde{V}^\dagger_{l'j'}(\omega^{'})\right\rangle = 0 \end{gathered} \tag{S.13b}$$

We define the amplitude and phase quadratures as follows: $\hat{X}_i = \hat{a}_i + \hat{a}_i{}^\dagger, \hat{Y}_i = -i\left(\hat{a}_i - \hat{a}_i{}^\dagger\right)$.

Below threshold, the mean values of the fields are zero. The fluctuations of the quadratures can be studied by analyzing the following linearized dynamics:

$$\dot{\begin{bmatrix}\hat{X}\\ \hat{Y}\end{bmatrix}} = J\begin{bmatrix}\hat{X}\\ \hat{Y}\end{bmatrix} + \sqrt{2\alpha}\begin{bmatrix}\hat{W}_0^\alpha\\ \hat{W}_{\pi/2}^\alpha\end{bmatrix} + \sqrt{2\mu}\begin{bmatrix}\hat{W}_0^\mu\\ \hat{W}_{\frac{\pi}{2}}^\mu\end{bmatrix} \tag{S.14}$$

where, $\hat{X} = [\hat{X}_1 \dots \hat{X}_N]^T$, $\hat{Y} = [\hat{Y}_1 \dots \hat{Y}_N]^T$, $\hat{W_i^j} = [W_0^{\hat{j},a_1} \dots W_0^{\hat{j},a_N}]$ for $j \in \{\mu, \alpha\}$ and $i \in \{0, \frac{\pi}{2}\}$.

$$J = -\gamma I_{2N} + g\begin{bmatrix}I_N & 0\\ 0 & -I_N\end{bmatrix} + \begin{bmatrix}0 & -H_L\\ H_L & 0\end{bmatrix} \tag{S.15}$$

$I_N$ is an identity matrix of order N. $I_{2N}$ is an identity matrix of order 2N. $J$ is matrix of 2N x 2N.

and, $\hat{W}_{j,0}^l = \hat{V}_{l,j} + \hat{V}_{l,j}^\dagger$, $\hat{W}_{j,\pi/2}^l = -\mathrm{i}\left(\hat{V}_{l,j} - \hat{V}_{l,j}^\dagger\right)$, $l \in \{\mu, \alpha\}$ and $j \in \{a_1 \dots a_N\}$. The respective output field can be mapped to the intra-cavity field as:

$$\begin{bmatrix}\hat{X}_{out}\\ \hat{Y}_{out}\end{bmatrix} = \sqrt{2\mu}\begin{bmatrix}\hat{X}\\ \hat{Y}\end{bmatrix} - \begin{bmatrix}\hat{W}_0^\mu\\ \hat{W}_{\frac{\pi}{2}}^\mu\end{bmatrix} \tag{S.16}$$

In the spectral domain we get,

$$\begin{bmatrix}\tilde{X}_{out}(\omega)\\ \tilde{Y}_{out}(\omega)\end{bmatrix} = -\sqrt{2\mu}\,[J + \mathrm{i}\omega I_{2N}]^{-1}\left(\sqrt{2\alpha}\begin{bmatrix}\hat{W}_0^\alpha(\omega)\\ \hat{W}_{\frac{\pi}{2}}^\alpha(\omega)\end{bmatrix} + \sqrt{2\mu}\begin{bmatrix}\hat{W}_0^\mu(\omega)\\ \hat{W}_{\frac{\pi}{2}}^\mu(\omega)\end{bmatrix}\right) - \begin{bmatrix}\hat{W}_0^\mu(\omega)\\ \hat{W}_{\frac{\pi}{2}}^\mu(\omega)\end{bmatrix} \tag{S.17}$$

The output correlation matrix can be written as:

$$C^{out}(\omega) = \int_{-\infty}^{\infty} d\omega' \left\langle \begin{bmatrix}\tilde{X}_{out}(\omega)\\ \tilde{Y}_{out}(\omega)\end{bmatrix}\begin{bmatrix}\tilde{X}_{out}(\omega')\\ \tilde{Y}_{out}(\omega')\end{bmatrix}^T\right\rangle \tag{S.18}$$

where $T$ stands for matrix transpose operation.

$$C^{out}(\omega) = \left(2\mu\,[J + \mathrm{i}\omega I_{2N}]^{-1} + I_{2N}\right) C^{in}(\omega)\left(2\mu\,[J - \mathrm{i}\omega I_{2N}]^{-1} + I_{2N}\right)^T + 4\mu\alpha\,(J + \mathrm{i}\omega I_{2N})^{-1} C^{in}(\omega)\left((J - \mathrm{i}\omega I_{2N})^{-1}\right)^T \tag{S.19}$$

The input correlation matrix is:

$$C^{in}(\omega) = \begin{bmatrix}I_N & \mathrm{i}I_N\\ -\mathrm{i}I_N & I_N\end{bmatrix} \tag{S.20}$$

# 6 Ring of Exceptional point

We explore 2D Kagome lattice of parametrically driven quadratic nonlinear resonators. If we vary the detuning of the resonators we can drive the bulk into PT broken regime. At the critical detuning, the eigenvalues become complex conjugate pairs. The eigenstates where the PT symmetry is broken occupies a ring like structure in the momentum space [3, 4]. At large values of detuning all the bulk modes are in the PT unbroken regime as shown in Fig. S7(a,b,c). At detuning values less than the critical detuning (that is characterized by the exceptional point), some of the eigenmodes undergo PT phase transition and assume imaginary eigenvalue with zero real part. This is shown in Fig. S7(d,e,f).

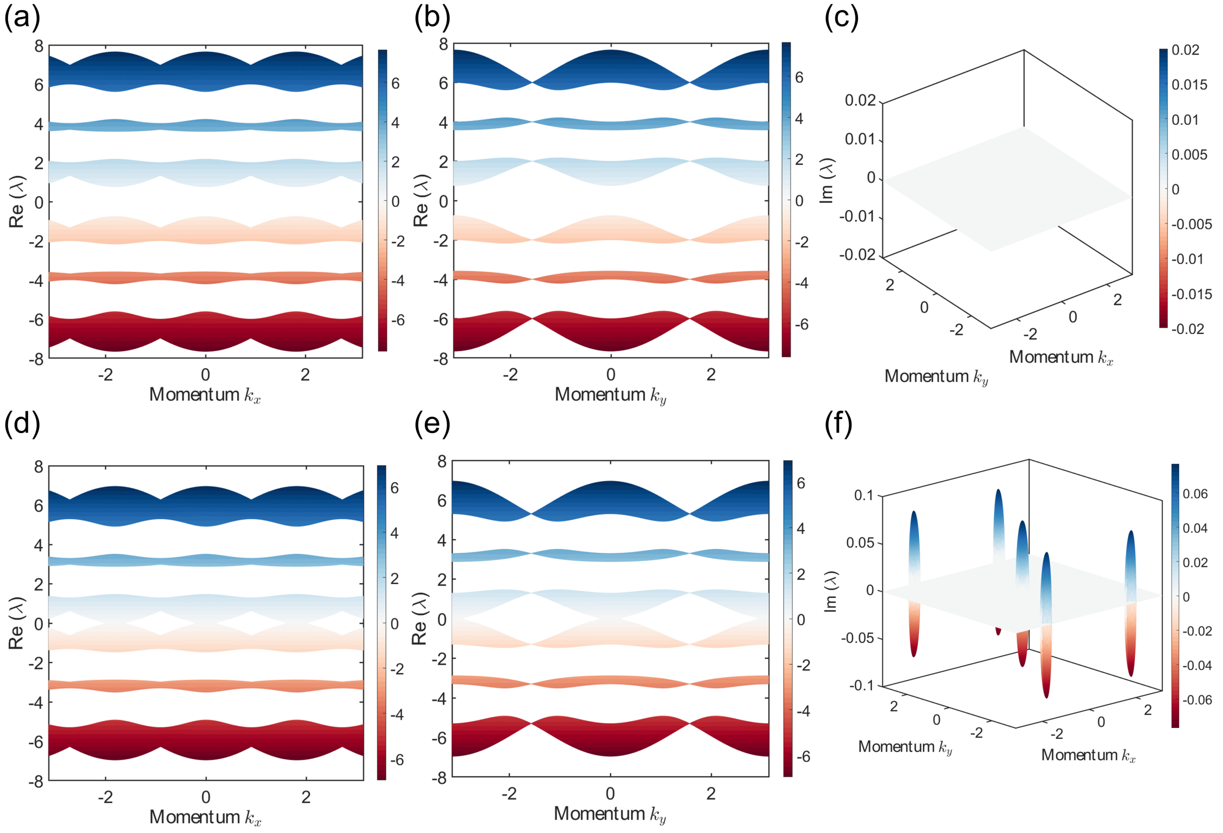


Figure S7: Band structure of the 2D lattice in the PT unbroken regime. a) real part projected in the XZ plane, b) real part projected in the YZ plane, c) the imaginary part. Parameters used in the simulation are: $\Delta = 4, \phi = 0.6\pi, g = 0.1, J = 1$. Band structure of the 2D lattice in the PT broken regime. d) real part projected in the XZ plane, e) real part projected in the YZ plane, f) the imaginary part. Parameters used in the simulation are: $\Delta = 3.3, \phi = 0.6\pi, g = 0.1, J = 1$.

# 7 Non-Hermitian transitions from the Imaginary part of the complex Berry phase

The complex Berry phase of a non-Hermitian Hamiltonian is given by [5]:

$$\phi = \oint \left\langle L(k) | \mathrm{i} \frac{d}{dk} R(k) \right\rangle dk \tag{S.21}$$

where the integral is calculated on a close path in the momentum space $[-\pi, \pi]$. $|R(k) >$ and $|L(k) >$ are the right and left eigenvectors of $\sigma_z$H(k) respectively. We just focus on $\phi_-$, which describes the Berry phase for the lower bulk band of the 1D SSH coupled system of OPOs.

$H(k)$ is given by:

$$H(k) = \begin{bmatrix} H_1(k) & H_2(k) \\ H_2(-k)^* & H_1(-k)^* \end{bmatrix} \tag{S.22}$$

$H_1(k)$ is expressed as:

$$H_1(k) = \begin{bmatrix} \Delta & \kappa_1 + \kappa_2 \mathrm{e}^{-\mathrm{i}k} \\ \kappa_1 + \kappa_2 \mathrm{e}^{\mathrm{i}k} & \Delta \end{bmatrix} \tag{S.23}$$

where $\Delta$ is the detuning, $\kappa_1 = J(1-\epsilon)$ and $\kappa_2 = J(1+\epsilon)$.

$H_2(k)$ is expressed as:

$$H_2(k) = \begin{bmatrix} g & 0 \\ 0 & g \end{bmatrix} \tag{S.24}$$

The right eigenvector of the lower band is given by: $|R(k)>= [-\frac{\rho}{g} + \sqrt{\frac{\rho}{\rho^*}}\frac{\Delta-\sqrt{(\Delta-|\rho|)^2-g^2}}{g}, -\frac{\Delta-|\rho|-\sqrt{(\Delta-|\rho|)^2-g^2}}{g}, -\sqrt{\frac{\rho}{\rho^*}}, 1]^T$.

where $\rho = \kappa_1 + \kappa_2 \mathrm{e}^{-\mathrm{i}k}$.

The left eigenvector of the lower band is given by: $|L(k)>= [\frac{\rho^*}{g} + \sqrt{\frac{\rho^*}{\rho}}\frac{-\Delta+\sqrt{(\Delta-|\rho|)^2-g^2}}{g}, -\frac{-\Delta+|\rho|+\sqrt{(\Delta-|\rho|)^2-g^2}}{g}, -\sqrt{\frac{\rho^*}{\rho}}, 1]^T$.

In Figure S8(a,d), we plot the imaginary part of the complex Berry phase as a function of the on-site detuning. The discontinuities in the derivative of the imaginary part of the Berry phase aligns with the underlying non-Hermitian phase transitions (Fig. S8(b,e)). This agrees with the trend of the imaginary part of the eigenvalues obtained from the numerical simulation of a finite 1D SSH lattice (Fig. S8(c,f)).

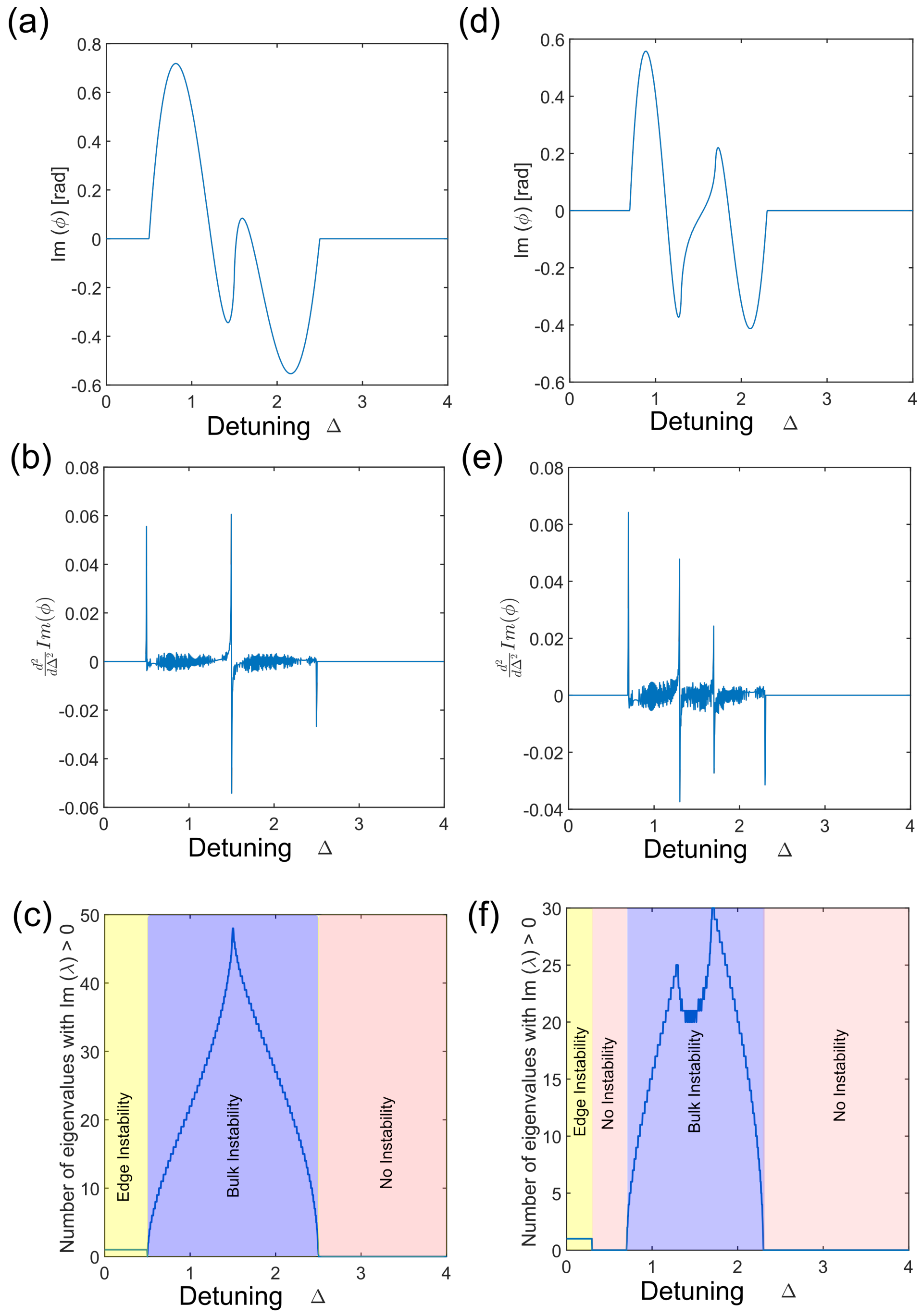


Figure S8: For g=0.5 a) Imaginary part of the complex Berry phase b) Double derivative of the imaginary part of the Berry phase, c) Imaginary part of the Eigenvalue distribution (Im ($\lambda$) 0) obtained numerically for a finite 1D SSH lattice for N=99. For g=0.3 d) Imaginary part of the complex Berry phase e) Double derivative of the imaginary part of the Berry phase, f) Imaginary part of the Eigenvalue distribution (Im ($\lambda$) 0) obtained numerically fr a finite 1D SSH lattice for N=99. Parameters used in the simulation are: $\epsilon = 0.5, J = 1$.

## 8 Topological protection of the edge mode eigenvalue in the presence of symmetry protected disorder

The eigenvalue corresponding to the edge mode in the 1D SSH coupled OPO arrays is not perturbed by small amount of symmetry preserving disorders. Here, the disorder is considered in the coupling co-coefficients ($\kappa_1$ and $\kappa_2$) which still preserves the chiral symmetry. The real part of the eigenvalue distribution is plotted in Fig. S9(a). We can see that the edge mode is not affected, while the bulk modes are affected by the introduction of the detuning disorder. The imaginary part of the eigenvalue distribution is shown in Fig. S9(b).

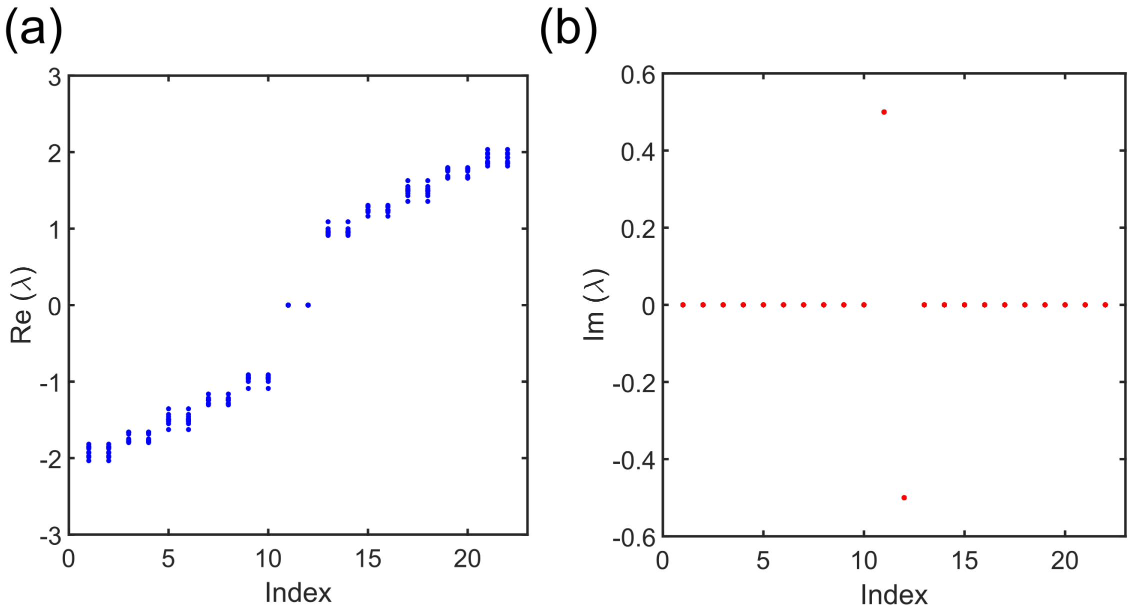


Figure S9: a) real part of the eigenvalue distribution of 1D SSH coupled OPO arrays. The edge mode is unperturbed by the disorder, while the bulk modes are affected by the disorder. b) imaginary part of the eigenvalue distribution. Parameters used in the simulation are: $\epsilon = 0.5, J = 1, N = 11, g = 0.5$. The coupling disorder is assumed to be normally distributed with standard deviation of 0.1. 10 different disorder realizations are overlaid on top of each other.

## 9 Numerical Simulation of topological parametric oscillation in the proposed system

The parametric interaction in the phase-matched periodically poled lithium niobate waveguide can be expressed in the slowly varying envelope approximation as [6]:

$$\frac{\partial A_\omega}{\partial z} = -\mathrm{i}\kappa A_{2\omega} A_\omega^* \tag{S.25a}$$

$$\frac{\partial A_{2\omega}}{\partial z} = -\mathrm{i}\kappa A_{\omega}^2 \tag{S.25b}$$

where, the electric fields are normalized such that the envelopes ($A_\omega$, $A_{2\omega}$) are expressed in $\mathrm{W}^{\frac{1}{2}}$.

The nonlinear coupling co-efficient ($\kappa$) can be expressed as: $\kappa = \frac{\sqrt{2Z_0}\omega d_{\text{eff}}}{cn_\omega\sqrt{n_{2\omega}A_{\text{eff}}}}$. Here, $n_\omega$ and $n_{2\omega}$ refers to the effective index of the fundamental TE mode at the signal and pump wavelengths respectively. $Z_0$ and $c$ represents the impedance of the free space and the velocity of light respectively. $d_{\text{eff}}$ is the effective nonlinear co-efficient, and $A_{\text{eff}}$ is the effective mode area.

The couplers can be modelled as an unitary matrix (M): $\begin{bmatrix} a_2 \\ b_1 \end{bmatrix} = \begin{bmatrix} \sqrt{1-r^2} & r \\ -r & \sqrt{1-r^2} \end{bmatrix} \begin{bmatrix} a_1 \\ b_2 \end{bmatrix}$ where, $a_1, b_2$ are the input amplitudes to the coupler, while $a_2, b_1$ are the output amplitudes.$r$ is the amplitude cross-coupling co-efficient. The field propagation sequentially experiences the nonlinear interaction, followed by adiabatic dichroic coupler, followed by pair of SSH coupling (only one for the case of edge OPOs), and finally through the other adiabatic dichroic coupler. The propagation loss is also taken into account.

The single OPO threshold is given by: $P_{th} = \left(\frac{\ln\left(\frac{1}{a(1-r^2)}\right)}{\kappa NL}\right)^2$. Here, $NL$ is the length of the periodically poled region responsible for the nonlinear interaction. $a^2 = \exp^{-\alpha L}$ is the round-trip absorption co-efficient due to propagation loss, and $L$ is the total round-trip length of the cavity. the factor of $1 - r^2$ is due to the loss of two dichroic couplers, one of which acts the output port as well.

Figure S10(a) depicts the schematic of a single OPO that constitutes the 1D SSH chain. The pump is non-resonant, while the signal is resonant and a part of it is out-coupled at the output port. The wavelength selective resonance behavior can be realized using an adiabatic dichroic coupler design [7, ?]. This will occupy the dashed rectangular region shown in the schematic. We consider parametric interaction between the signal at 1550 nm, and the pump at 775 nm. The parametric interaction occurs in the periodically poled region which is assumed to be 4 mm long. Inhomogeneities in the required poling periods in different OPOs in the 1D SSH lattice can be corrected via local integrated thermo-optic heaters. We assume an X-cut lithium niobate on silica platform, with a thin-film width of 600 nm. The waveguides are assumed to be 1.8 $\mu m$ wide, with an etch depth of 400 nm. The waveguides support TE modes at both the signal and pump wavelengths as shown in Fig. S10(b). The expected second harmonic generation efficiency is around 3500 %/W/cm$^2$. We assume an intrinsic quality factor of 1 million, and a loaded quality factor of approximately 750k, corresponding to a through power coupling of 2.5% at the dichroic couplers. This leads to a doubly-resonant OPO threshold of approximately 80 $\mu$W operating at degeneracy.

In order to realize a 1D SSH lattice the OPOs have to be coupled with each other [8]. This can be executed via evanescent couplers, like the ones shown in Fig. S10(c). We assume power coupling of 10% corresponding to $\kappa_1$, and coupling of 40% corresponding to $\kappa_2$. This will translate to varying the gap between the waveguides, considering the coupling length is fixed. When all the OPOs are pumped with 400 $\mu$W of pump power, it leads to topological parametric oscillation, where the lattice oscillates at the edge state as shown in Fig. S10(d). Power can be delivered simultaneously to multiple OPOs using fiber arrays mounted on V-grooves. Additionally, detuning mismatch between OPOs can be potentially corrected using integrated electro-optic modulators. At large values of parametric gain (for example at pump powers of 30 mW, that agrees with our theoretical calculations), bulk oscillation is favoured over edge-state oscillation.

We know from our analysis, that for the parameter values given by $g > 2\epsilon$, bulk instability occurs.

This translates to pump power of approximately 12.8 mW. The presence of passive loss (propagation + out-coupling) will slightly modify this threshold power for phase transition. This is shown in Fig. S11.

Fig. S12(a) shows representative schematic of the proposed SSH lattice. Asymmetric coupling can be realized by altering the spacing between the evanescent coupling based directional couplers. Another, possible layout is shown in Fig. S12(b).

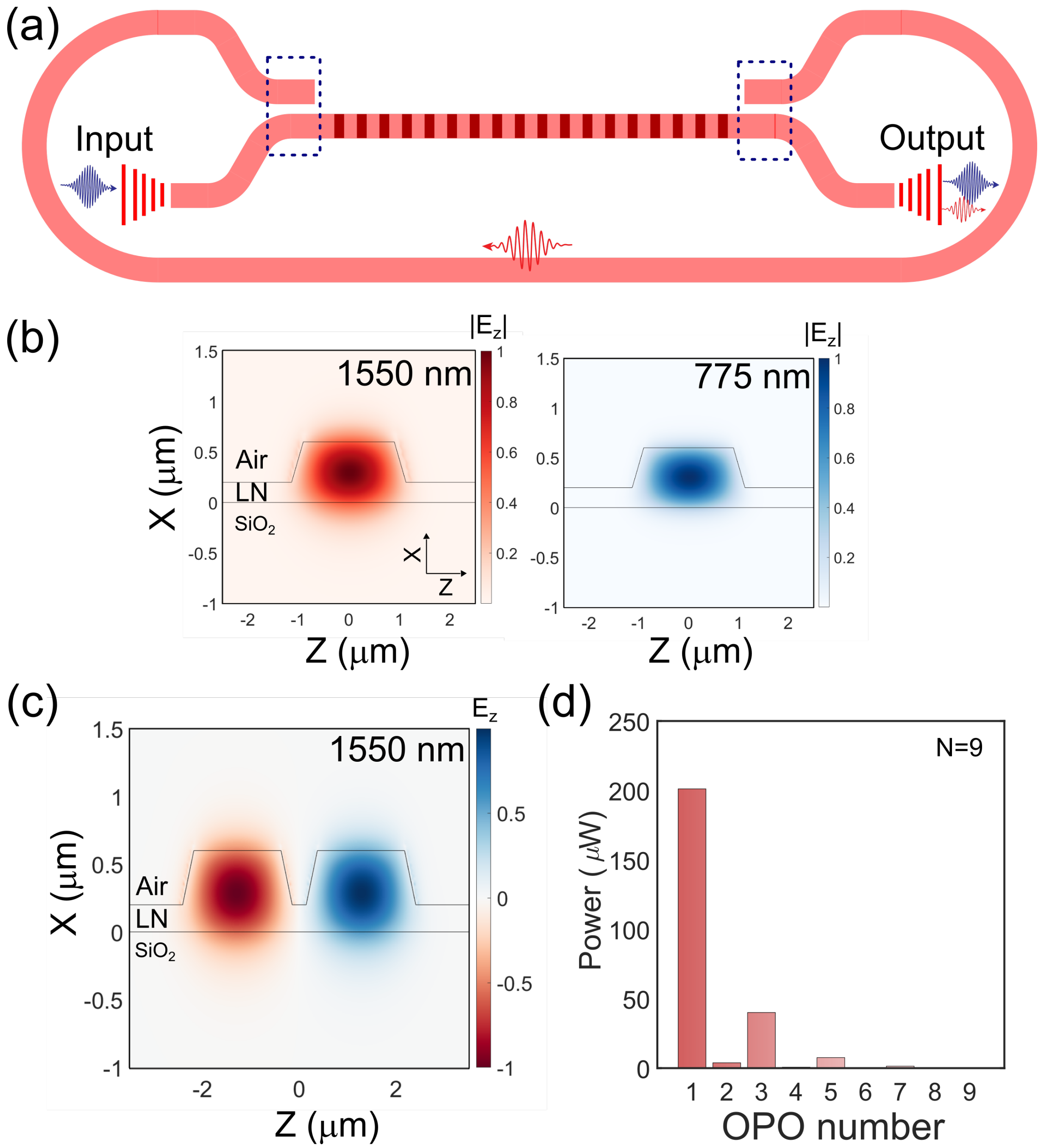


Figure S10: **Proposed system for the realization of topological optical parametric oscillation.** a) Schematic of an integrated OPO on thin-film lithium niobate that can form the constituent block of an 1D SSH chain. b) Electric field profiles for the fundamental TE modes supported by the waveguide at the signal and pump wavelengths. c) Coupling between adjacent OPOs can be realized via evanescent coupling between waveguides. Anti-symmetric mode supported in such a coupler is shown. d) Signal power distribution in each OPOs comprising the SSH lattice (N=9), as obtained from the numerical simulation, depicting the edge state oscillation.

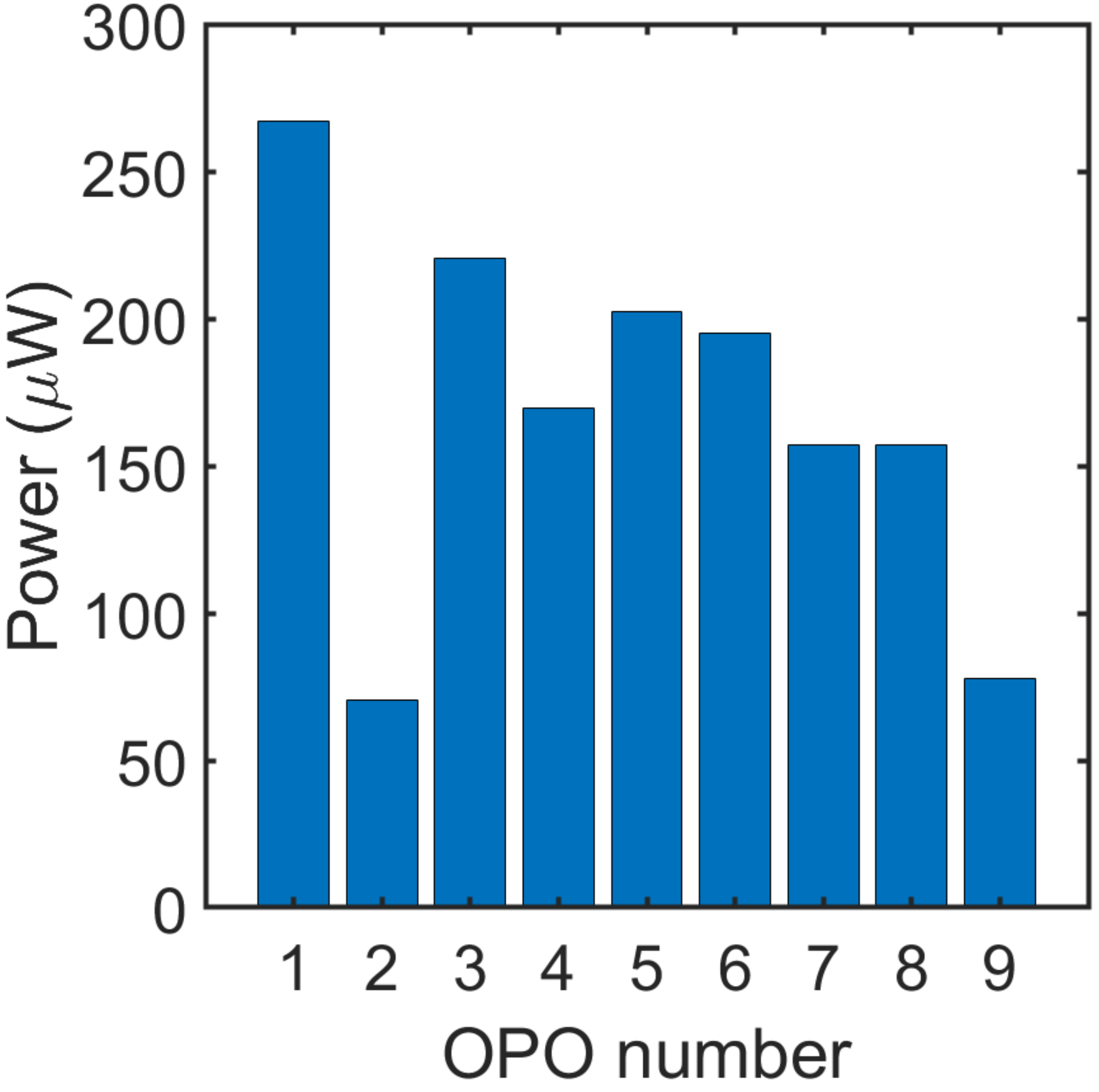


Figure S11: The system of OPOs oscillate in the bulk mode when $g > 2\epsilon$. In the simulation, the pump power for each OPO is assumed to be 30 mW.

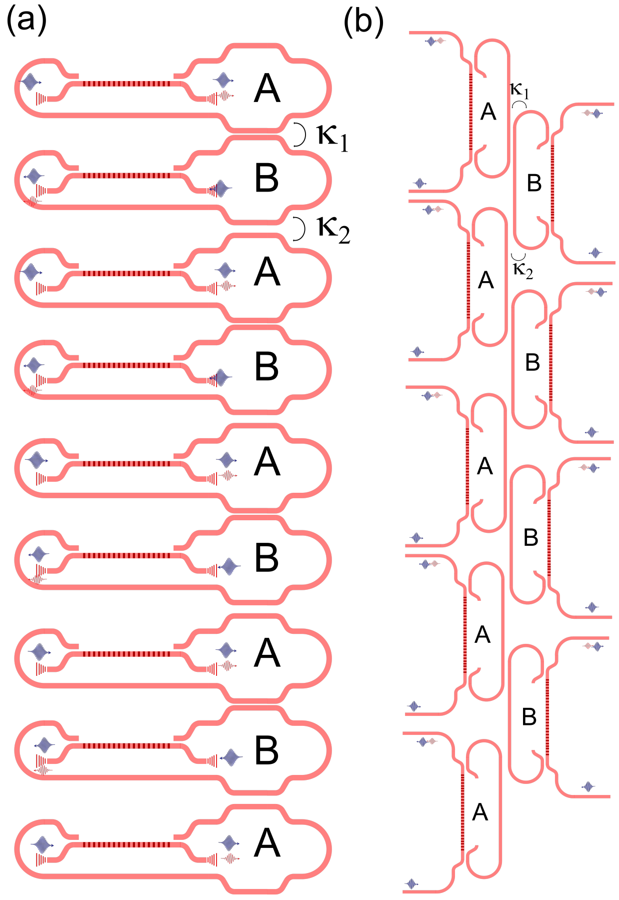


Figure S12: The schematic of SSH lattice of OPOs (N=9). The dimensions are not to scale. Two possible layouts are presented.

# References


[1] Gaurav Chaudhary, Michael Levin, and Aashish A Clerk. Simple approach to characterizing band topology in bosonic pairing hamiltonians. *Physical Review B*, 103(21):214306, 2021.

[2] Yanne K Chembo. Quantum dynamics of Kerr optical frequency combs below and above threshold: Spontaneous four-wave mixing, entanglement, and squeezed states of light. *Physical Review A*, 93(3):033820, 2016.

[3] Alex Y Song, Xiao-Qi Sun, Avik Dutt, Momchil Minkov, Casey Wojcik, Haiwen Wang, Ian AD Williamson, Meir Orenstein, and Shanhui Fan. P t-symmetric topological edge-gain effect. *Physical Review Letters*, 125(3):033603, 2020.

[4] Tsuneya Yoshida and Yasuhiro Hatsugai. Exceptional rings protected by emergent symmetry for mechanical systems. *Physical Review B*, 100(5):054109, 2019.

[5] Shi-Dong Liang and Guang-Yao Huang. Topological invariance and global berry phase in non-hermitian systems. *Physical Review A*, 87(1):012118, 2013.

[6] Marc Jankowski, Carsten Langrock, Boris Desiatov, Alireza Marandi, Cheng Wang, Mian Zhang, Christopher R Phillips, Marko Lončar, and MM Fejer. Ultrabroadband nonlinear optics in nanophotonic periodically poled lithium niobate waveguides. *Optica*, 7(1):40–46, 2020.

[7] Xiang Guo, Chang-ling Zou, Carsten Schuck, Hojoong Jung, Risheng Cheng, and Hong X Tang. Parametric down-conversion photon-pair source on a nanophotonic chip. *Light: Science & Applications*, 6(5):e16249–e16249, 2017.

[8] Abhi Saxena, Yueyang Chen, Zhuoran Fang, and Arka Majumdar. Photonic topological baths for quantum simulation. *ACS Photonics*.

[9] Arkadev Roy, Saman Jahani, Qiushi Guo, Avik Dutt, Shanhui Fan, Mohammad-Ali Miri, and Alireza Marandi. Nondissipative non-hermitian dynamics and exceptional points in coupled optical parametric oscillators. *Optica*, 8(3):415–421, 2021.

[10] Midya Parto, Steffen Wittek, Hossein Hodaei, Gal Harari, Miguel A Bandres, Jinhan Ren, Mikael C Rechtsman, Mordechai Segev, Demetrios N Christodoulides, and Mercedeh Khajavikhan. Edge-mode lasing in 1d topological active arrays. *Physical review letters*, 120(11):113901, 2018.
